# Motavizumab, A Neutralizing Anti-Respiratory Syncytial Virus (Rsv) Monoclonal Antibody Significantly Modifies The Local And Systemic Cytokine Responses Induced By Rsv In The Mouse Model

**DOI:** 10.1186/1743-422X-4-109

**Published:** 2007-10-25

**Authors:** Asunción Mejías, Susana Chávez-Bueno, Martin B Raynor, John Connolly, Peter A Kiener, Hasan S Jafri, Octavio Ramilo

**Affiliations:** 1 Department of Pediatrics, Division of Pediatric Infectious Diseases, University of Texas Southwestern Medical Center at Dallas, Texas, USA; 2 Baylor Institute for Immunology and Research at Dallas, Texas, USA; 3 MedImmune Inc, Gaithersburg, MD, USA

## Abstract

Motavizumab (MEDI-524) is a monoclonal antibody with enhanced neutralizing activity against RSV. In mice, motavizumab suppressed RSV replication which resulted in significant reduction of clinical parameters of disease severity. We evaluated the effect of motavizumab on the local and systemic immune response induced by RSV in the mouse model. Balb/c mice were intranasally inoculated with 10^6.5 ^PFU RSV A2 or medium. Motavizumab was given once intraperitoneally (1.25 mg/mouse) as prophylaxis, 24 h before virus inoculation. Bronchoalveolar lavage (BAL) and serum samples were obtained at days 1, 5 (acute) and 28 (long-term) post inoculation and analyzed with a multiplex assay (Beadlyte Upstate, NY) for simultaneous quantitation of 18 cytokines: IL-1α, IL-1β, IL-2, IL-3, IL-4, IL-5, IL-6, KC (similar to human IL-8), IL-10, IL-12p40, IL-12p70, IL-13, IL-17, TNF-α, MCP-1, RANTES, IFN-γ and GM-CSF. Overall, cytokine concentrations were lower in serum than in BAL samples. By day 28, only KC was detected in BAL specimens at low concentrations in all groups. Administration of motavizumab significantly reduced (p < 0.05) BAL concentrations of IL-1α, IL-12p70 and TNF-α on day 1, and concentrations of IFN-γ on days 1 and 5 compared with RSV-infected untreated controls. In the systemic compartment, the concentrations of IL-10, IFN-γ and KC were significantly reduced in the motavizumab-treated mice compared with the untreated controls. In summary, prophylactic administration of motavizumab was associated with significant reductions on RSV replication and concentrations of cytokine and chemokines, which are likely related to the improvement observed in clinical markers of disease severity.

## Findings

Respiratory Syncytial Virus (RSV) is the main viral respiratory pathogen causing hospitalization in infants and young children worldwide[1]. It infects nearly 70% of infants in their first year of life and almost all children by the age of two [2]. The mechanisms by which RSV causes pulmonary disease and more specifically which factors determine disease severity still remains to be fully characterized. It is increasingly appreciated that symptoms and signs of RSV are caused not only by the direct viral cytopathic effect but also by the host response to infection. Nonetheless, in RSV disease both viral replication and the exaggerated immune response to RSV infection are closely interrelated. In fact, studies suggest that the pattern of cytokine production elicited by RSV affects the balance between virus replication and disease pathogenesis, that ultimately determines the manifestations of the disease [3].

In the present study we took an alternative approach to explore the relative importance and role that different cytokines and chemokines play in acute RSV disease severity.

Instead of targeting individual cytokines as potential therapeutic targets, we took advantage of our experience with motavizumab. We previously showed that the superior neutralizing activity of this anti-RSV monoclonal antibody compared with palivizumab was associated with further reductions in RSV replication which in turn resulted in additional improvement in clinical disease severity [4,5]. The present study was designed to assess the effect of motavizumab on the cytokine and chemokine responses induced by RSV both in the respiratory tract and in the systemic compartment, which, we hypothesized, were likely associated with the observed improvement in disease severity.

Seven-week-old female, pathogen-free BALB/c mice (Charles River) were intranasally inoculated with 100 μL of 10^6.5 ^PFU RSV-A2 or sterile 10% Eagle's minimal essential medium, following institutional guidelines [5-7]. Motavizumab was administered intraperitoneally 24 h before RSV inoculation (1.25 mg in 0.1 ml of PBS/per mouse) [5]. Our previous studies showed that no treatment or treatment with either PBS or an IgG1 isotype-matched control antibody, MEDI-507, at the same time of the administration of the anti-RSV antibody had no effect on the cytokine profile or other clinical and inflammatory parameters evaluated, therefore those controls were not included in the study [8]. Bronchoalveolar lavage (BAL) and serum samples from 4–6 mice per time point/group from two independent experiments were obtained during the acute, (days 1 and 5 post-inoculation) and chronic (day 28) phases of the disease. Paired BAL and serum samples from non-infected controls, RSV-infected untreated, and RSV-infected mice treated with motavizumab previously stored at -80°C, were randomly selected within the two experiments (4 mice per time-point/group) for cytokine analysis using the Beadlyte Mouse Multi-Cytokine Detection System (Upstate Biotechnology, Lake Placid NY) and the Luminex^100 ^plate reader (Luminex Corporation, Austin, TX) according to manufacturer's instructions. Quantification of cytokines was performed by regression analysis from a standard curve generated from cytokine standards included in the kit with a lower limit of detection of 10 pg/ml for all cytokines evaluated. The plaque assay, which has a lower limit of detection of 1.7 log_10 _PFU/mL, was used to measure RSV viral loads in BAL specimens as previously described [5-7]. Disease severity was assessed by whole-body plethysmograph (Buxco, Troy, NY) to evaluate airway obstruction (AO) by measuring the enhanced pause [7-9]. According to data distribution, Mann-Whitney rank sum test or t-test were used for analyses. Since the concentrations of the cytokines/chemokines evaluated were detected either at a very low level or were below the level of detection of the assay in the non-infected control group, and the objective of the study was to evaluate the effect of motavizumab in the inflammatory response induced by RSV, the uninfected control group was not included in the statistical analyses.

**Figure 1:**
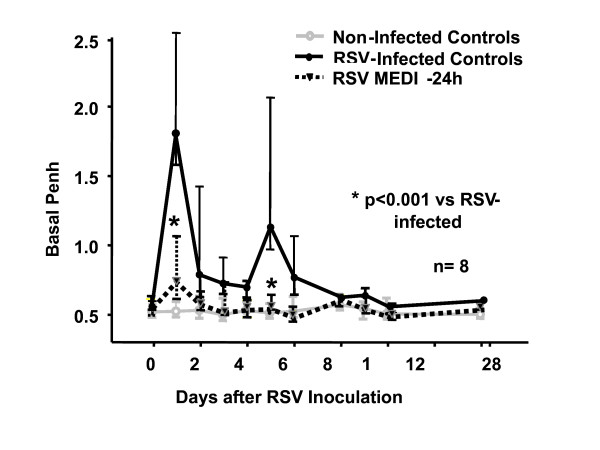
**Effect of the anti-RSV mAb (motavizumab) on pulmonary function**. Airway obstruction was assessed by whole body plethysmograph by measuring basal Penh daily during the first two weeks after infection and weekly up to day 28. Values represent median; errors bars, 25^th^–75^th ^percentile. Each group consisted of 8 mice. Results of two separate experiments are shown. * p < 0.001 by Kruskal-Wallis ANOVA on ranks for comparison between RSV-infected untreated, sham inoculated controls and RSV-infected treated with motavizumab at -24 h.

Motavizumab prophylaxis completely prevented the development of clinical disease objectively assessed by measuring the AO; in fact, mice treated with the mAb remained clinically asymptomatic throughout the experiment compared with the RSV-infected untreated mice (Figure 1). Furthermore, motavizumab administration resulted in significant reductions of RSV loads compared with untreated controls on days 1 and 5 (< 1.7 PFU/ml log_10 _vs 2.45 [2.22–2.53] on day 1 and 3.12 [2.73–3. 39] on day 5; p < 0.05).

Eighteen cytokines, including interleukins (IL)-1α, -1β, -2, -3, -4, -5, -6, KC (similar to human IL-8), IL-10, -12p40/70, -13, -17, tumor necrosis factor-α (TNF-α), macrophage chemoattractant protein-1 (MCP-1), Regulated upon Activation, Normal T-cell Expressed and Secreted (RANTES), interferon-γ (IFN-γ) and granulocyte macrophage colony-stimulating factor (GM-CSF) were measured using a multiplex antibody assay. Except for IL-10, MCP-1, IL-1β and IL-12p70 concentrations, the effect of motavizumab on the cytokine profile was more evident in the respiratory tract (BAL) than in serum. This could be explained in part because fewer cytokines were detected in serum and also because the concentrations were lower in serum than in BAL specimens.

On day 1, BAL concentrations of IL-1α, IL-12p70, TNF-α and IFN-γ and serum IL-10 and KC were significantly lower in mice treated with motavizumab compared with RSV-infected untreated mice (Table 1). The role of TNF-α in RSV disease is not completely understood. While some authors have suggested that TNF-α has a protective role in RSV infection, others have related the RSV-induced lung damage to the overproduction of TNF-α [10,11]. In our previous studies, palivizumab administration did not modify BAL concentrations of TNF-α nor the initial peak of airway obstruction (AO) observed in this model [8]. In contrast, motavizumab significantly reduced BAL concentrations of TNF-α, and this was unexpectedly associated with suppression of the initial peak of AO on day 1. Although the pathogenesis of the first peak of AO in this model is not completely understood, this is the first time that we observed its suppression, suggesting that early production of TNF-α may play a major role in initiating airway disease.

**Table 1:** BAL/serum cytokine concentrations (pg/mL) in RSV infected mice treated with motavizumab compared with untreated RSV infected controls on day 1 after inoculation. Each group consisted of 4 samples randomly selected from two independent experiments.

	**BAL (D1)**			**SERUM (D1)**		
				
	**RSV Untreated**	**RSV Motavizumab**	**p value**	**RSV Untreated**	**RSV motavizumab**	**p value**
**IFN-γ**	67.16 ± (24.6)	28.01 ± (13.8)	**0.03**	ND	ND	---
**MCP-1**	1930.68 ± (716.6)	1088.12 ± (307.8)	0.07	381.83 (341.0–554.8)	230.65 (217.43–298.83)	0.6
**RANTES**	1952.10 ± (999.05)	671.66 ± (340.4)	0.051	ND	ND	---
**IL-10**	39.29 ± (27.0)	14.47 ± (7.8)	0.1	102.23 ± (22.5)	16.78 ± (13.56)	**0.001**
**TNF-α**	2290.90 ± (1100.2)	279.03 ± (204.7)	**0.01**	ND	ND	---
**GM-CSF**	101.42 (45.7–144.9)	25.14 (22.1–34.0)	0.1	ND	ND	---
**IL-12p70**	44.14 ± (13.5)	22.59 ± (8.7)	**0.03**	45.85 ± (17.5)	23.75 ± (16.1)	0.16
**KC**	3967.43 (2039.8–5795.3)	1303.57 (946.4–1657.1)	0.1	215.86 ± (125.8)	37.63 ± (32.5)	**0.03**
**IL-6**	2580.89 ± (1413.6)	821.79 ± (290.13)	0.051	91.39 (45.43–135.31)	31.17 (18.97–35.85)	0.2
**IL-1β**	477.52 (231.53–831.92)	182.78 (179.4–214.3)	0.3	266.88 ± (74.4)	119.70 ± (143.5)	0.16
**IL-1α**	289.75 ± (114.0)	76.28 ± (29.1)	**0.01**	ND	ND	---
**IL-17**	ND	ND	---	20.68 (11.94–31.85)	10.67 (10.06–12.03)	0.2

**Note**: Data are shown as mean ± SD, or medians and 25–75% range as appropriate according to whether they were normally distributed. ND (non-detected)

Our previous studies showed that tracheal aspirate concentrations of RANTES, IL-8 and IL-10 in children intubated with severe RSV inversely correlated with clinical disease severity, however these cytokines were not measured in serum [12]. Interleukin-10, an anti-inflammatory/regulatory cytokine, has been shown not only to be an important component of the pathogenesis of acute RSV bronchiolitis but to also play a role in the enhanced airway hyperreactivity that occurs after RSV infection [13,14]. In our study, motavizumab significantly decreased serum concentrations of KC (chemoattractant for neutrophils, the predominant lung inflammatory cell during acute RSV) and IL-10, two cytokines that play a major role in the pathogenesis of RSV bronchiolitis [13]. BAL concentrations of IL-6, RANTES and MCP-1 were also decreased in the motavizumab group, but likely due to small numbers there was insufficient power to achieve statistical significance (Tables 1, 2).

**Table 2:** BAL/serum cytokine concentrations in RSV infected mice treated with motavizumab compared with untreated RSV infected controls on day 5 after inoculation. Each group consisted of 4 samples randomly selected from two independent experiments.

	**BAL (D5)**			**SERUM (D5)**		
				
	**RSV Untreated**	**RSV Motavizumab**	**p value**	**RSV Untreated**	**RSV Motavizumab**	**p value**
**IFN-γ**	1291.29 ± (561.3)	640.84 ± (388.8)	**0.02**	116.58 ± (43.1)	28.29 ± * (35.7)	**0.02**
**MCP-1**	291.10 ± (128.6)	132.0 ± (105.8)	0.15	311.95 ± (78.3)	343.26 ± (81.2)	0.6
**RANTES**	118.82 ± (28.3)	48.50 ± (51.9)	0.055	ND	ND	---
**IL-10**	59.7 ± (52.91)	25.68 ± (18.5)	0.3	15.18 (10.0–71.9)	23.28 (15.18–48.82)	0.6
**TNF-α**	13.65 (10.32–17.35)	10.0 (10.0–11.18)	0.2	ND	ND	---
**IL-12p70**	ND	ND	---	39.72 ± (20.6)	38.07 ± (5.8)	0.7
**IL-8 (KC)**	209.9 ± (88.8)	117.75 ± (54.2)	0.12	32.54 ± (27.96)	25.82 ± (19.6)	0.7
**IL-6**	101.38 ± (51.22)	30.46 ± (28.87)	0.052	ND	ND	ND
**IL-1β**	34.63 ± (34.59)	52.25 ± (54.3)	0.6	180.66 ± (87.03)	126.09 ± (58.3)	0.4
**IL-1α**	12.59 ± (3.05)	13.84 ± (3.7)	0.6	ND	ND	ND
**IL-17**	ND	ND	---	13.02 (10.0–22.98)	10.61 (10.0–11.77)	0.5

**Note**: Data are shown as mean ± SD, or medians and 25–75% range as appropriate according to whether they were normally distributed. ND (non-detected).

On day 5, the peak of viral replication and lung inflammation in this model, both serum and BAL concentrations of INF-γ were significantly decreased in the motavizumab group (Table 2). To date, there is still no consensus on the role of INF-γ in RSV pathogenesis and its responses may vary depending on disease severity [15-17]. Mice treated with motavizumab had no virus detected by culture in the respiratory tract, (< 1.7 PFU/mL log_10_) and this reduction in virus load was associated with significantly decreased local and systemic IFN-γ concentrations, and more importantly with no evidence of AO in these mice suggesting a pivotal role of INF-γ in the pathogenesis of RSV disease. The fact that substantial IFN-γ was detected on day 5 despite treatment with the mAb suggests that some viral particles still escaped the effect of the enhanced neutralizing antibody. This interpretation of the results is further supported by our previous experiments in which UV-inactivated virus did not induce an INF-γ response [7].

By day 28, only KC was detected in BAL specimens at low concentrations without significant differences between groups.

Taking together, these results suggest that not only the local but also the systemic cytokine immune response influence the severity of acute RSV disease, and that RSV clinical disease can be modified with the use of a specific anti-RSV neutralizing antibody directed against a well conserved epitope of the RSV F protein.

In summary, in the mouse model, prophylactic administration of motavizumab significantly decreased RSV replication, the local and systemic cytokine responses, especially TNFα, IL-1α, IL-12p70, KC, IL-10 and INF-γ, and completely prevented the development of clinical acute RSV disease. Future studies in humans treated with anti-RSV antibodies should determine whether changes in clinical markers of disease severity correlate with similar changes in cytokine profiles. These studies will provide further understanding of the pathogenesis of RSV infection and should help identifying new targets for developing immunomodulatory therapies.

## Competing interests

O.R. and H.J. serve as members of the Medimmune Pediatric Infectious Disease Advisory Board. OR, HJ and AM have received research grants from Medimmune and P.K. is an employee of Medimmune.

## Authors' contributions

A.M study design, data analyses, manuscript preparation; SCB data analyses and manuscript review, and MBR performing experiments; JC Luminex data analysis and interpretation. PK and HSJ data interpretation; OR project design, experimental analyses and interpretation, manuscript preparation.
